# Co-optimization Learning Network for MRI Segmentation of Ischemic Penumbra Tissues

**DOI:** 10.3389/fninf.2021.782262

**Published:** 2021-12-16

**Authors:** Liangliang Liu, Jing Zhang, Jin-xiang Wang, Shufeng Xiong, Hui Zhang

**Affiliations:** ^1^College of Information and Management Science, Henan Agricultural University, Zhengzhou, China; ^2^Department of Computer Science, Henan Quality Engineering Vocational College, Pingdingshan, China; ^3^Department of Computer Science, University of Melbourne, Parkville, VIC, Australia

**Keywords:** co-optimization learning network, ischemic penumbra tissues, segmentation network, reconstruction network, transfer block

## Abstract

Convolutional neural networks (CNNs) have brought hope for the medical image auxiliary diagnosis. However, the shortfall of labeled medical image data is the bottleneck that limits the performance improvement of supervised CNN methods. In addition, annotating a large number of labeled medical image data is often expensive and time-consuming. In this study, we propose a co-optimization learning network (COL-Net) for Magnetic Resonance Imaging (MRI) segmentation of ischemic penumbra tissues. COL-Net base on the limited labeled samples and consists of an unsupervised reconstruction network (R), a supervised segmentation network (S), and a transfer block (T). The reconstruction network extracts the robust features from reconstructing pseudo unlabeled samples, which is the auxiliary branch of the segmentation network. The segmentation network is used to segment the target lesions under the limited labeled samples and the auxiliary of the reconstruction network. The transfer block is used to co-optimization the feature maps between the bottlenecks of the reconstruction network and segmentation network. We propose a mix loss function to optimize COL-Net. COL-Net is verified on the public ischemic penumbra segmentation challenge (SPES) with two dozen labeled samples. Results demonstrate that COL-Net has high predictive accuracy and generalization with the Dice coefficient of 0.79. The extended experiment also shows COL-Net outperforms most supervised segmentation methods. COL-Net is a meaningful attempt to alleviate the limited labeled sample problem in medical image segmentation.

## 1. Introduction

### 1.1. Clinical Motivation and Challenges

**Figure 1:**
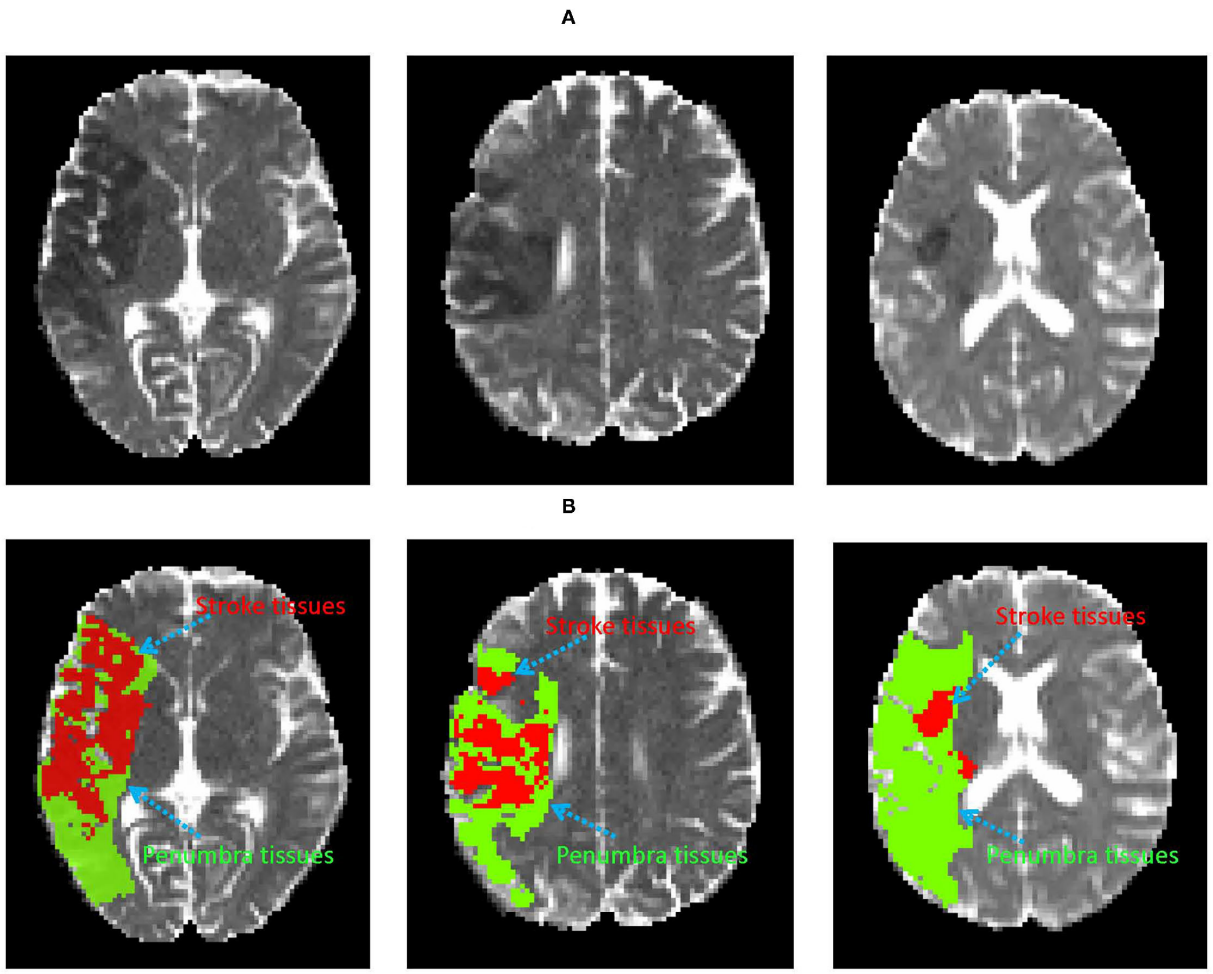
Examples of the infarct core and penumbra tissues on DWI image **(A)**. The red and green area denote infarct core and penumbra tissues **(B)**, respectively.

Labeling target tissue from medical images is of great significance for disease diagnosis and treatment. Recently, the application of computer technology in brain image tissue segmentation has become a hot field of medical imaging analysis (Lutnick et al., 2019; Huseyn, 2020; Zhao et al., 2020). Brain penumbra is a common affliction of ischemic stroke diseases in men. Ischemic penumbra segmentation on magnetic resonance image (MRI) is important for stroke diagnosis and pre-operative planning (Dora et al., 2017; Maier et al., 2017; Liu et al., 2020b). In medical physiology, the penumbra tissue is introduced to designate regions of brain tissue with “almost ischemia" (Lassen et al., 1991). The infarct region of stroke has been necrotic, while the penumbra tissues can be saved. Accurate annotation of penumbra tissues will help clinicians to locate the penumbra tissues and make an effective individualized therapy. As shown in Figure 1A, the dark area is the infarct tissue of stroke, it is surrounded by penumbra and normal tissues. The treatment of stroke disease is mainly based on vascular recanalization in an effective time window. In other words, it is to save the penumbra tissues and restore the penumbra tissues to normal tissue through intravenous thrombosis and intravascular treatment. As shown in Figure 1B, the penumbra tissues are usually distributed around the infarct tissues. Accurate annotation of penumbra tissues plays a key role in taking active measures to save penumbra tissues or transform them into normal tissues. Unfortunately, consistent and correct labeling of penumbra tissues based on MRI is challenging for experienced doctors, due to 1) unclear tissue boundaries, 2) differences in subjective experience 3) boring and time-consuming work.

Computer technology (image processing, machine learning, deep learning, etc.) has been applied to brain image analysis. Especially the supervised segmentation convolutional neural networks (CNNs) can provide the possibility for medical image lesion segmentation. Some supervised deep learning methods have shown advantages in many segmentation tasks. However, a fine segmentation model still faces some challenges: 1) The lack of the massive labeled sample is difficult to meet the demand of a supervised deep learning method to train a fine model. Supervised segmentation models are hard to achieve the feature representation on the limited labeled data set. 2) Rough and non-uniform manual labels impair the ability of the pedication model to mine the key hidden features of the target tissues. 3) The accuracy of semi-supervised and unsupervised segmentation methods is not satisfactory. In the case of limited labeled samples, how to develop a segmentation method to improve the efficiency of delineation of unlabeled samples is our concern.

### 1.2. Related Work

Deep learning is the most representative technology in computer vision (Hu et al., 2018, 2019; Zhang et al., 2021; Zhou et al., 2021). Many deep learning-based studies have been applied in medical image segmentation tasks in past decades (Dora et al., 2017; Liu et al., 2020b; Hu et al., 2021). These studies are mainly divided into two types: the methods based on supervised learning strategy and the methods based on semi-supervised learning strategy.

The supervised learning strategy trains models automatically to improve the learning performance by using labeled samples (Knudsen, 1994). The supervised CNNs are representative of this kind of strategy. In the training process, CNNs learn the highly representative hidden features under the guidance of the labeled samples. To a certain extent, these methods promote the development of medical image segmentation (Moeskops et al., 2018; Liu et al., 2019, 2020a; Nakarmi et al., 2020). For example, the Ischemic Stroke Lesion Segmentation (ISLES) 2015 challenge showed that the supervised segmentation methods could obtain good segmentation results on stroke MRI images (Maier et al., 2017). Kamnitsas et al. Kamnitsas et al. (2016) proposed a dual pathway 3D supervised CNN for brain tumor segmentation in BraTS 2015 challenge. In 2017, Esteva et al. developed a supervised deep neural network for analyzing skin cancer, which greatly inspired the research in skin cancer analysis (Andre et al., 2019). In addition, supervised learning strategies are used to improve the performance of image analysis models, including: spine cancer (Esteva et al., 2017), optic disc (Fu et al., 2018), liver and prostate (Litjens et al., 2014; Li et al., 2018), etc. The supervised learning strategy improves the accuracy of the model, however, the limited sample in the medical image analysis task is an unavoidable challenge to the supervised segmentation models. These models can not utilize the extra information from unlabeled samples.

The semi-supervised learning strategy trains models automatically to improve the learning performance by using labeled samples and unlabeled samples (Van Engelen and Hoos, 2020). Semi-supervised learning aims to improve the performance of the prediction model by learning from a limited number of labeled data and an arbitrary amount of unlabeled data. This strategy is more suitable for medical image analysis with limited samples and assists clinicians to delineate unlabeled samples (Weston et al., 2012; He et al., 2020; Li et al., 2020a). For example, Li et al. proposed a semi-supervised model for medical image segmentation (Li et al., 2020b), they optimized the model by using two loss functions: public supervision loss from labeled samples and regularization loss from unlabeled samples. They introduced a transformation-consistent strategy to enhance the regularization effect for pixel-level predictions in the semi-supervised segmentation tasks. The proposed model showed superior performance on the 3 challenging 2D/3D medical image data sets. Liu et al. provided a relation-driven semi-supervised framework for medical image classification (Liu et al., 2020e). The framework is a consistency-based method. Firstly, this method used unlabeled samples by encouraging the prediction consistency of a given input under disturbance and then used self-scrambling model to generate high-quality consistency targets for unlabeled samples. This method outperformed many state-of-the-arts semi-supervised learning methods on ISIC 2018 challenge and thorax disease classification with Chest X-ray images. Kumar et al. integrated the idea of semi-supervised learning into the model for retinopathy and cancer anomaly detection (Kumar and Awate, 2020). This model achieved high-quality outlier labeling by a small amount of expert calibration data. Kumar et al. adopted the maximum semi-supervised robust hybrid model strategy to improve the detection performance of the model. In addition, as a special case of semi-supervised learning, few-shooting learning is proposed to solve the problem of the limited labeled samples (Fei-Fei et al., 2006; Vinyals et al., 2016). Shaban et al. (2017) proposed a conditioning branch and segmentation branch network to solve the semantic segmentation problem on limited samples. The whole network could predict the type of a test image at the pixel level. Rakelly et al. proposed a train of guided and conditional networks with few labeled samples and sparse annotations (Rakelly et al., 2018). This approach was evaluated on the PASCAL VOC computer vision benchmark. These methods usually used unlabeled samples to assist models to make a prediction. However, these methods are easily affected by pixel values with large differences in the medical image. Especially in low contrast brain images, semi-supervised based methods usually mistakenly regard the infarct area as the optimization target in stroke imaging segmentation task, which will result in the interference of the training process and the degradation of model performance.

### 1.3. Contributions

In this study, we present a semi-supervised learning network (COL-Net) based on co-optimization learning for brain image segmentation. COL-Net can be trained by jointly using the limited labeled and unlabeled MRI images. The whole network is co-optimized by combining the supervised and unsupervised sub-networks (loses). To leverage the unlabeled samples, we assume each labeled sample also has a pseudo unlabeled state. We take the advantage of co-optimization learning works into delineating penumbra tissues. Based on the limited number of labeled and pseudo unlabeled samples, we leverage pseudo unlabeled samples as additional information for the segmentation model and optimize the model. Our contributions include:

(1) We propose a co-optimization learning network for ischemic penumbra tissues segmentation which is an attempt to assist clinicians to delineate penumbra tissues under the limited labeled samples.

(2) COL-Net consists of 3 parts: segmentation network (S), reconstruction network (R), and transfer block (T). R is used as the auxiliary branch of S with the pseudo unlabeled samples, S is used to predict the results with the limited labeled samples and the supervise of R, and T is used to co-optimize the feature maps between the bottlenecks of R and S.

(3) We use a mix loss function (segmentation loss and reconstruction loss) to co-optimize COL-Net.

(4) Extensive experiments and ablation study demonstrates that COL-Net substantially outperforms well-tuned state-of-the-art methods.

The rest of this paper is organized as follows. The methodology of the proposed network is introduced in section 2. Section 3 introduces the materials and provides the evaluation metrics of our segmentation network. We introduce the experimental setup and analyze the experimental results in detail. Section 5 conducts and discusses the contributions of individual components in COL-Net. Finally, we make the conclusions of this study in section 6.

## 2. Methodology

### 2.1. The Architecture of COL-Net

**Figure 2:**
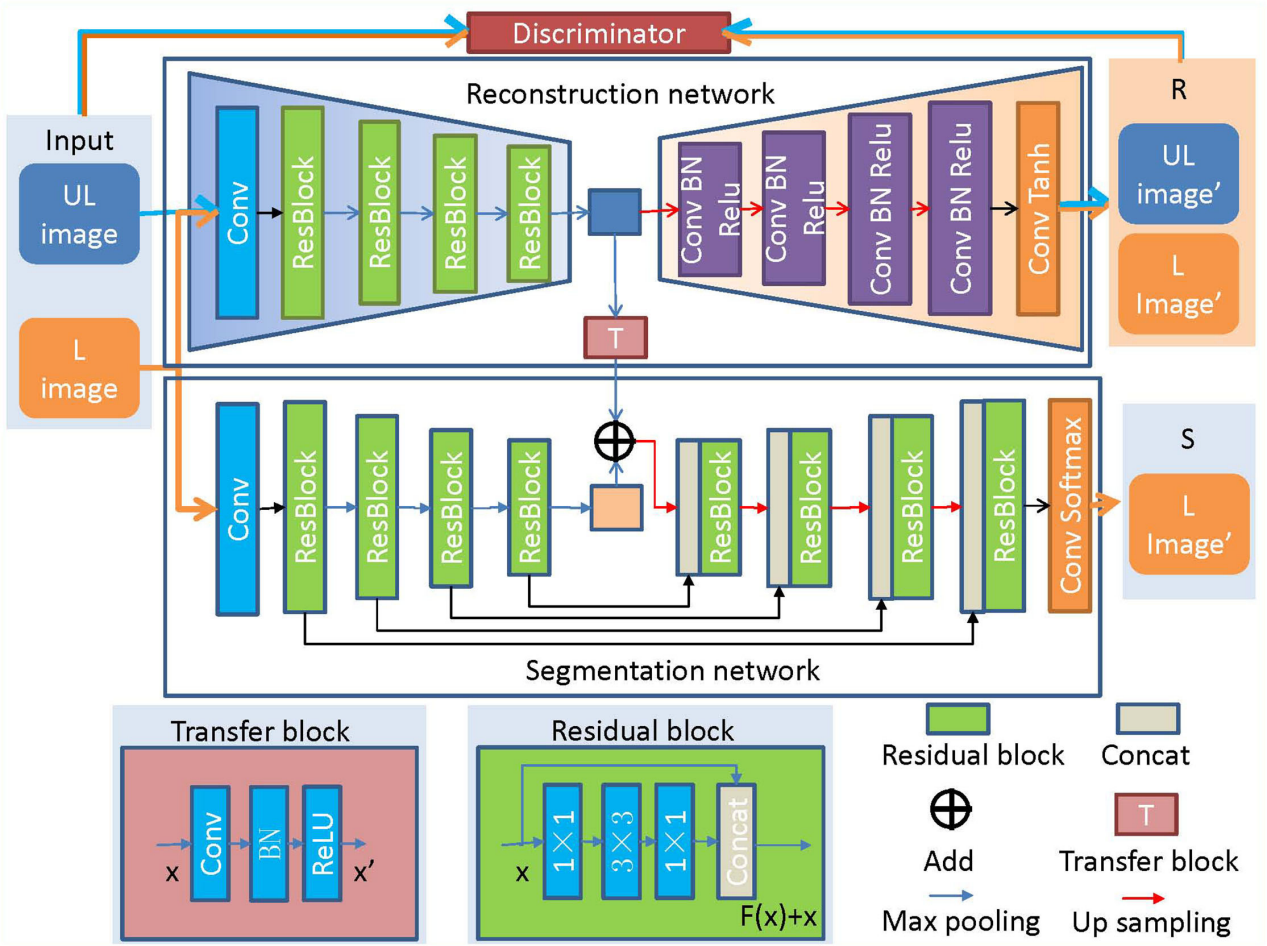
The architecture of COL-Net. “UL”: unlabeled image; “L”: labeled image; “R”: reconstruction network; “S”: segmentation network; “T”: transfer block.

The architecture of COL-Net is shown in Figure 2 and Algorithm 1 summarizes the pseudocode. COL-Net mainly consists of 3 parts: an unsupervised reconstruction network (R), a supervised segmentation network (S), and a transfer block (T).

**Algorithm 1:** COL-Net Algorithm pseudocode.

**Input:** $x_{i}\in L,y_{i}\in L,x_{i}^{\prime }\in UL$
**Output:** ${\tilde{X}}_{k}$
1: $R\left(x_{i}|x_{i}^{\prime }\right)\leftarrow$ unsupervised reconstruction network
2: *S*(*x*_*i*_)← supervised segmentation network
3: *featrure*_*R*_← middle layer in $R\left(x_{i},x_{i}^{\prime }\right)$
4: *featrure*_*S*_← bottleneck layer in *S*(*x*_*i*_)
5: *T*(*R, S*)←*add*(*featrure*_*R*_, *featrure*_*S*_)
6: **for** *i* = 1 to numepochs **do**
7: randomly update $R\left(x_{i}|x_{i}^{\prime }\right),S\left(x_{i}\right)$
8: $loss_{r}\left(x_{i}|x_{i}^{\prime }\right)\leftarrow$ update loss $\left(R\left(x_{i}|x_{i}^{\prime }\right)\right)$
9: *loss*_*s*_(*x*_*i*_)← update loss (*S*(*x*_*i*_))
10: *loss*_*mix*_←*min*(*loss*_*r*_+Λ*loss*_*s*_)
11: **end for**
12: **return** *loss*_*mix*_

### 2.2. Network Architecture

#### 2.2.1. The Encoder-Decoder Framework

The encoder-decoder architecture is a common framework in deep learning (Long et al., 2015; Ronneberger et al., 2015; Liu et al., 2020b,d). It can be used to analyze any text, voice, image, and video data. The encoder-decoder architecture is an end-to-end learning algorithm and usually is used as the backbone of CNN, RNN, BiRNN, LSTM, GRU, and so on. The encoder converts the input sequence into a fixed-length vector. The decoder converts the previously generated fixed vector into the output sequence. In medical image analysis tasks, residual or dense mechanisms, and skip connection operation are usually embedded into the encoder-decoder framework to improve the model performance (Ronneberger et al., 2015; He et al., 2016; Zhang et al., 2018).

In our study, the unsupervised network (R) and supervised network (S) are the main parts of COL-Net, both of them adopt the encoder-decoder architecture as the backbone. COL-Net is trained in a co-optimization manner: 1) We train R by using the pseudo unlabeled samples to obtain representative features. 2) We joint the feature maps of R and S as the input of up-sampling by transfer block and further use the labeled samples to fine-tune the S.

#### 2.2.2. Reconstruction Network (R)

**Figure 3:**
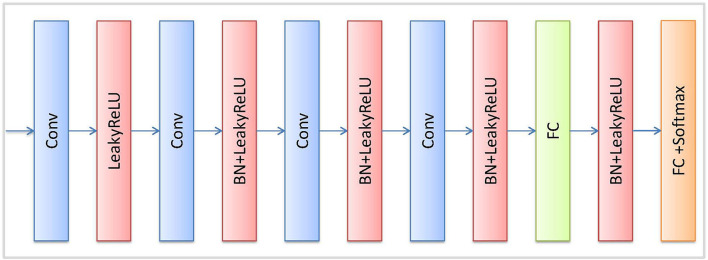
Detailed architecture of discriminator block.

The reconstruction network (R) is trained in the unsupervised strategy. R is illustrated in Figure 2, the encoder part of R comprises followed that in ResNet network (He et al., 2016). The decoder part of R adopts 4 continuous Conv-BN-Relu (Conv: convolution, BN: batch normalization, and ReLU: rectified linear unit) blocks to reconstruct the feature map. Finally, a convolution layer and *Tanh* function are used to reconstruct the image. We also use a discriminator network (D) to distinguish the input image from the generated image by the reconstruction network. The architecture of D is illustrated in Figure 3, which contains 4 convolutional layers with a 3 × 3 kernel, a fully connected (FC) layer with 512 neurons, and an FC layer followed by the *softmax* activation function.

We aim at improving the segmentation performance for the COL-Net, reconstruction network plays as an auxiliary and correction network of the segmentation network in COL-Net. Reconstruction network is trained under the pseudo unlabeled samples in an unsupervised way. Given the *i* − *th* input sample *X*_*i*_, *i* ∈ [1, *I*], the loss function of R is defined as follow:

(1) $$\begin{array}{r} loss_{r}\left(X_{i}\right)=loss_{mse}\left(R\left(X_{i}\right),X_{i}\right)-\\ {}[loss_{ce}(D(X_{i}),0)+loss_{ce}(D(X_{i}),1)], \end{array}$$

where *loss*_*mse*_() is the mean square error (MSE) loss (Masci et al., 2011), which measures the discrepancy between the original image and the reconstructed image. The *loss*_*ce*_() is the cross-entropy adversarial loss for D, which measures the discrepancy between the distributions of an input image and the reconstructed one. We use the Adam optimizer to fine optimize the *loss*_*r*_() loss function.

#### 2.2.3. Segmentation Network (S)

As illustrated in Figure 2, the segmentation network is inspired by the encoder-decoder architecture and is trained under the supervised strategy. The encoder-decoder architecture has been proved to perform well in medical image segmentation tasks (Long et al., 2015; Ronneberger et al., 2015). We construct the backbone of the segmentation network (S) based on ResNet (He et al., 2016). The encoder in S is the same as that in R. In addition, the skip connections make the feature mapping and fuse the feature mappings between the encoder layer and decoder layer, and the continuous up-samplings are used to recognize feature and gradually recover the location of each pixel. Finally, we use the *softmax* activation function to predict the segmentation result. The detail of the residual block is shown in the subgraph of Figure 2, it consists of 3 convolution layers (1 × 1, 3 × 3, 1 × 1) and a fusion layer (*Concat*()).

We use the labeled samples to train the segmentation network. The loss function (*loss*_*s*_) of the segmentation network bases on the Dice coefficient (DC) (Milletari et al., 2016). In the medical image analysis task, DC is usually based on the pixel level, it is calculated the similar proportion between the prediction result and the label image. The larger the DC value, the better the segmentation result. Given the *j*−*th* labeled sample *X*_*j*_ and corresponding label *Y*_*j*_, where *j* ∈ [1, *J*], the *loss*_*s*_ comes from DC, which is defined as follows:

(2) $$\begin{array}{r} loss_{s}=1-DC\left(X_{j},Y_{j}\right)=1-\frac{2|Y_{j}⋂X_{j}|}{\left|Y_{j}|+|X_{j}\right|}, \end{array}$$

where *DC* is a measure of similarity between image *X*_*j*_ and label *Y*_*j*_.

In our study, we hope the reconstruction network can supervise the segmentation network to achieve the aim of using the features of the unlabeled sample to assist segmentation. In our model, we use a mixed loss function to train and co-optimize COL-Net. The mixed loss function is based on the segmentation network and the reconstruction network. The proposed COL-Net is co-optimized during the training process, the optimizing strategy compose of two parts: *loss*_*r*_ and *loss*_*s*_. The *loss*_*r*_ is trained on the pseudo unlabeled samples in an unsupervised manner. The *loss*_*s*_ is trained on the labeled samples in a supervised manner. We use a mix loss function to train COL-Net by reducing to the minimum of the following function:

(3) $$\begin{array}{l} loss_{mix}=\min\left(l_{s}+\lambda l_{r}\right)\\ =\min(1-\frac{2|Y_{j}⋂X_{j}|}{\left|Y_{j}|+|X_{j}\right|}\\ -\lambda l_{mse}\left(R\left(X_{i}\right),X_{i}\right)-\left[l_{ce}\left(D\left(X_{i}\right),0\right)+l_{ce}\left(D\left(X_{i}\right),1\right)\right]) \end{array}$$

where λ is employed to weight the importance of the reconstruction network. It should be noted that when *l*_*s*_ is set to 0, the parameter λ should be set to 0 to ensure the training process of the model.

#### 2.2.4. Transfer Block (T)

**Figure 4:**
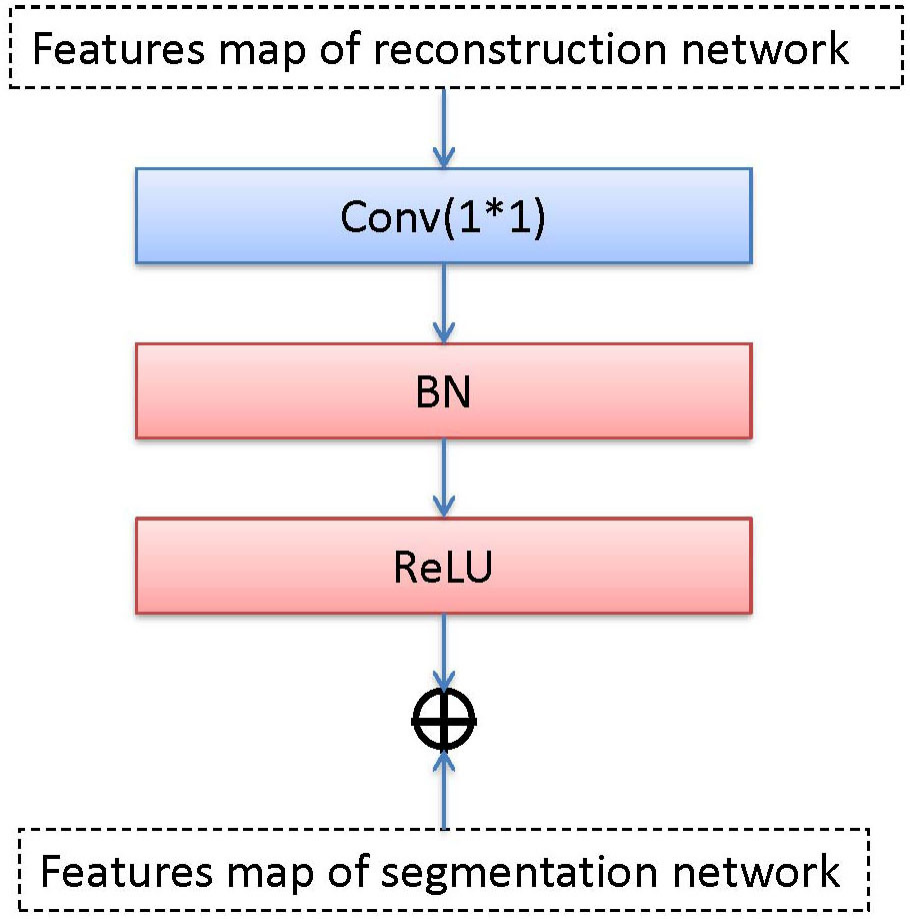
Detailed pipeline of transter block.

The transfer block is used as a bridge between R and S, which transfers the features maps by the convolution, BN, and ReLU operations. We transfer the output of the middle layer in R and fuse it with the output of the bottleneck layer in S. The transfer block is illustrated in Figure 4, the T block is a 1 × 1 convolutional layer with a stride of 1 followed by BN and ReLU activation.

We use *add*() method to fuse two feature maps that come from two bottleneck layers (the reconstruction network and the segmentation network). *add*() superimposes pixel values. It is a pixel information overlay method, which increases the amount of information describing the features of an image, the channel information in the image does not increase. Each feature map is fused according to channels. Let ∗ be the convolution operation and *f*^*i*^ be the *i*-th feature map. *f*^*i*^ consists of channels $A_{1}^{i}$, $A_{2}^{i}$, …, $A_{c}^{i}$. There has *n* feature maps. The equation of the output feature map with *add*() can be expressed as:

(4) $$\begin{array}{l} O_{add}=\overset{c}{\underset{j\;=\;1}{\sum }}\left(A_{j}^{1}*K_{j}^{1}+\ldots +A_{j}^{n}*K_{j}^{n}\right)\\ =\overset{c}{\underset{j\;=\;1}{\sum }}A_{j}^{1}*K_{j}^{1}+\ldots +\overset{c}{\underset{j\;=\;1}{\sum }}A_{j}^{n}*K_{j}^{n}, \end{array}$$

where *K* is the convolution kernel, *c* is the number of channels.

## 3. Evaluation Dataset

### 3.1. SPES Image Data

The Ischemic Stroke Lesion Segmentation (ISLES) launched a challenge to estimate the regions of the penumbra in acute stroke in 2015 (SPES). SPES challenge provides 50 acute ischemic stroke participants (30 for training and 20 for testing). All participants had been diagnosed and collected by the University Hospital of Bern between 2005 and 2013. All participants are older than 18. There are 7 MRI modalities for each participant: T1 contrast-enhanced (T1c), T2, DWI, cerebral blood flow (CBF), cerebral blood volume (CBV), time-to-peak (TTP), and time-to-max (Tmax). All MRIs were performed on either a 1.5T Siemens Magnetom Avanto or a 3T MRI system Siemens Magnetom Trio. All MRI modalities were skull-stripped, re-sampled to an isotropic spacing of 2^3^*mm*, and co-registered to the T1w contrast modality.

The ground truth of training samples are manually labeled in DWI images by a medicine doctor who obeyed the following steps: First, medicine doctor obtained the fusion restriction label region through a semi-manual segmentation method based on the Tmax modality. Then, he obtained the diffusion restriction label region through the currently accepted ADC threshold (600 × 10^−6^*mm*^2^/*sec*). Finally, the penumbra region was obtained by the mismatch of these two labels which were defined as follows: (*perfusion* − *restrictionlabel*) − (*diffusion* − *restrictionlabel*). The ground truth of training samples are provided on the challenge web page^1^. The ground truths of testing samples are not available on the challenge web page.

### 3.2. Evaluation Metrics

To demonstrate the advantage of COL-Net, we inherit the evaluation metrics of the SPES challenge and compare it with the supervised methods in the challenge and other supervised \ semi-supervised methods. SPES challenge offers 3 evaluation metrics: DC, Hausdorff distance (HD) (Huttenlocher et al., 1993), and Average Symmetric Surface Distance (ASSD). The DC is used to assess the similar proportion of overlap between the predicted image and the ground truth, DC is defined in Equation (3). (0 ≤ *DC* ≤ 1, the higher the DC value, the better the segmentation result). The HD is used to quantify the maximum distance between two surface pixels between the predicted image and ground truth. It is defined as follows:

(5) $$\begin{array}{r} HD\left(X,Y\right)=\max\left\{\underset{x\in X}{\max}\underset{y\in Y}{\min}d\left(x,y\right),\underset{y\in Y}{\max}\underset{x\in X}{\min}d\left(y,x\right)\right\}, \end{array}$$

where *X* and *Y* are predicting image and ground truth, respectively. *x* and *y* are the pixels in *X* and *Y*, and *d*(.) is the Euclidean distance between points *x* and *y*. The HD values are expressed in millimeters (*mm*).

The ASSD is used to evaluate the average distance between predict image and ground truth on pixel level, which is defined as:

(6) $$\begin{array}{l} ASSD\left(X,Y\right)=\frac{1}{2}(\frac{\sum_{x\in X}min_{y\in Y}d\left(x,y\right)}{\left|X\right|}\\ \;+\;\frac{\sum_{y\in Y}min_{x\in X}d\left(y,x\right)}{\left|Y\right|}), \end{array}$$

where the ASSD values are expressed in millimeters (*mm*). For HD and ASSD evaluation measures, the smaller the value is, the better is the segmentation results.

## 4. Experiments and Results

### 4.1. Experimental Setup

The SPES dataset provides 30 labeled training samples and 20 unlabeled testing samples. The reconstruction network is trained in an unsupervised manner and the segmentation network is trained in a supervised manner. In the training process, according to two different scenarios, we assume that each training sample has two label states: labeled and pseudo unlabeled samples. The labeled samples are used to train the segmentation network, the pseudo unlabeled samples are used to train the reconstruction network, the testing samples are just used to test the model. In the details, we use all pseudo unlabeled samples to train the reconstruction network and use 5 different percentages of labeled samples to train the segmentation network: 10% labeled samples (3 labeled training samples), 20% labeled samples (6 labeled training samples), 50% labeled samples (15 labeled training samples), 80% labeled samples (24 labeled training samples), 100% labeled samples (30 labeled training samples).

Under the unsupervised manner, the reconstruction network is optimized by the Adam optimizer, the mini-batch is 3, the epoch is 300, the learning rate is 10^−5^, the drop-out rate is 10^−2^, and the random weight initialization. We use the early stop strategy to train COL-Net and save the best weight of the model. In the segmentation network, the labeled training samples are randomly split into two parts: training part and validation part. In our study, all experiments are work on the sample level. We use the validation split method to divide the training and validation samples. The value of split parameter is set of 0.2. The training samples are used to train the models, the validation samples are used to fine-tune the trained models. Although segmentation and reconstruction networks have the same architecture, the initialization parameters of segmentation network are different from that of the reconstruction network. The segmentation network uses Adam function as an optimizer, the mini-batch size is 3, the epoch is 80, the learning rate is 10^−4^, the drop-out rate is 10^−2^, and the random weight initialization. Both networks work on the same computer server with an NVIDIA GeForce Titan X Pascal CUDA GPU with 16 GB memory. We save the best weight parameters of the segmentation model by maximizing the performance of the training process. And then we use the best model weight parameters to predict the segmentation results on the 20 testing samples.

### 4.2. Result and Analysis

In order to obtain the best semi-supervised segmentation model, we evaluate 5 training strategies respectively. In these 5 models, the last model (COL-Net5: using 100% labeled samples in the training process) is the only one under the supervised training strategy, others are under the semi-supervised training strategy. For each model, we test the best weight parameters on the 20 testing samples and submit the predicted results to the SPES challenge web page. These 5 predicated scores of testing samples are shown in Table 1. From COL-Net1 to COL-Net5, the results of the proposed COL-Net achieve relatively consistent improvements on 3 metrics. When the proportion of labeled samples used for training is less than 50%, the segmentation result is not satisfactory. When the number of labeled samples for training reaches 80% (COL-Net4), the DC value of COL-Net4 can achieve 79% which is the same as the DC value of using 100% labeled samples model (COL-Net5), which demonstrates that the reconstruction network provides the useful information for the segmentation network. It denotes that our proposed the co-optimization learning strategy has the effectiveness on limited labeled samples.

**Table 1:** The results of different strategies on the SPES dataset.

**Method**	**DC**	**ASSD**	**HD**
COL-Net1 (10%) (Semi)	37.00 (±12.00)	9.86 (±7.53)	76.57 (±43.17)
COL-Net2 (20%) (Semi)	53.00 (±9.00)	7.23 (±5.40)	62.61 (±32.40)
COL-Net3 (50%) (Semi)	68.00 (±12.00)	4.00 (±4.01)	59.30 (±25.91)
COL-Net4 (80%) (Semi)	79.00 (±8.00)	2.41 (±2.19)	**34.20** (±24.05)
COL-Net5 (100%) (Supe)	**79.00** (±9.00)	**1.83** (±0.52)	39.20 (±25.38)

*DC:%, ASSD:mm, HD:mm. Semi, Semi-supervised training strategy; Supe, Supervised training strategy. The bold values mean the best results*.

### 4.3. Ranking in the Challenge

All the top ranking methods are supervised methods in the SPES challenge web page. We use the COL-Net model which is obtained by the third training strategy to verify the testing samples (the only 80% of the labeled training samples are used in the segmentation network). We compare COL-Net with other top 7 participate teams in the SPES challenge. The rankings of these 7 teams are frozen by the ISLES workshop^2^. The top 7 predicated results of the test dataset on SPES are shown in Table 2. Compared with these supervised methods, COL-Net ranks the third. COL-Net obtains DC score is 79.00%, the ASSD is 2.41 *mm*, and the HD is 34.20 *mm*. CH-Insel obtains the best DC score (82.00%) which is higher than that of COL-Net 3.00%. DE-UzL obtains the best ASSD (1.36 *mm*) and HD (23.62 *mm*) scores which are only 1.05 *mm* and 10.58 *mm* lower (better) than that of COL-Net. Although our model does not achieve the best ranking, as a semi-supervised segmentation method, the result of COL-Net is a very competitive in all supervised methods.

**Table 2:** The results of the participants on the SPES dataset.

**Methods**	**DC**	**ASSD**	**HD**
CH-Insel (Supe)	**82.00** (±8.00)	1.65 (±1.40)	29.02 (±12.60)
DE-UzL (Supe)	81.00 (±9.00)	**1.36** (±0.74)	**23.62** (±12.99)
COL-Net (Semi)	79.00 (±8.00)	2.41 (±2.19)	34.20 (±24.05)
BE-Kul2 (Supe)	78.00 (±9.00)	2.77 (±3.27)	40.27 (±25.10)
CN-Neu (Supe)	76.00 (±9.00)	2.29 (±1.76)	30.65 (±16.49)
DE-Ukf (Supe)	73.00 (±13.00)	2.44 (±1.93)	33.92 (±20.88)
BE-Kul1 (Supe)	67.00 (±24.00)	4.00 (±3.39)	57.95 (±28.77)
CA-USher (Supe)	54.00 (±26.00)	5.53 (±7.59)	59.89 (±30.18)

*DC:%, ASSD:mm, HD:mm. Semi, Semi-supervised training strategy; Supe, Supervised training strategy. The bold values mean the best results*.

### 4.4. Comparison With the State-of-the-Art Methods

In addition, we also compare COL-Net with the state-of-the-art methods: U-Net (Ronneberger et al., 2015), FCN (Long et al., 2015), Res-UNet (He et al., 2016), Res-FCN (Liu et al., 2018), RA-UNet (Jin et al., 2018), and DRANet (Liu et al., 2020c). These methods are all supervised methods. We either use the code released by the authors or re-implement them exactly as described by the authors. To ensure the fairness of the comparison, we adopt multi-modalities MRI as input including DWI, CBV, Tmax, CBF, T2, and TTP as the inputs of all comparison methods. The training samples and testing samples of all supervised methods follow the SPES challenge and the evaluation scores come from the official challenge web page.

Table 3 shows the comparison results of the DC, ASSD, and HD scores of these 7 methods. Among all the methods, U-Net, FCN, Res-FCN, Res-UNet, RA-UNet, and DRANet are supervised methods. These methods use the residual mechanism or attention mechanism as the key to improve the segmentation performance of the methods. U-Net and FCN only use the end-to-end structure as the main backbone of the framework. Based on U-shape structure, the residual mechanism is added to Res-FCN and Res-UNet. RA-UNet and DRANet embed residual mechanism and attention mechanism into the encoder-decoder structure. These methods are under the supervised training strategy, they obtain the context information mainly come from the labeled samples, without the help of external auxiliary unlabeled samples. In these methods, RA-UNet and DRANet obtain the best results on 3 metrics: DC scores of 80.00–80.00%, ASSD value of 2.03–1.91 *mm*, and HD value of 39.43–30.13 *mm*. Both methods obtain more supplementary semantic information from attention mechanisms. The attention mechanisms help both methods extract the spatial features and focus on spatial semantic information. Compared with these supervised methods, COL-Net obtains a DC score is 79.00%, the ASSD is 2.41 *mm*, and the HD is 34.20 *mm*. Although the segmentation results of COL-Net are not great as that of RA-UNet and DRANet, however, under the co-optimization learning strategy and using limited samples to obtain competitive results is a meaningful attempt to assist medical image labeling.

**Table 3:** The results of the state-of-the-arts methods on the 2D SPES images.

**Methods**	**DC**	**ASSD**	**HD**
U-Net (Liang et al., 2017)	68.00 (±16.00)	3.49 (±2.77)	51.86 (±24.93)
FCN (Long et al., 2015)	70.00 (±12.00)	3.54 (±2.04)	60.42 (±28.56)
Res-FCN (Liu et al., 2018)	71.00 (±12.00)	3.43 (±1.94)	61.05 (±23.75)
Res-UNet (He et al., 2016)	76.00 (±9.00)	2.10 (±0.57)	42.54 (±21.14)
COL-Net	79.00 (±8.00)	2.41 (±2.19)	34.20 (±24.05)
RA-UNet (Jin et al., 2018)	80.00 (±3.00)	2.03 (±0.86)	39.43 (±27.07)
DRANet (Liu et al., 2020c)	80.00 (±7.00)	1.91 (±1.02)	**30.13** (±20.32)

*DC:%, ASSD:mm, HD:mm. The bold value mean the best results*.

## 5. Discussion

The good performance of supervised deep learning mainly depends on the training of a large number of labeled data. In this study, we are committed to developing a semi-supervised co-optimization learning network for brain image segmentation, which uses unlabeled data to improve the performance of the segmentation model and reduce the labeling workload.

### 5.1. Reconstruction Network

In this study, we propose a co-optimization learning framework COL-Net for penumbra tissues segmentation. COL-Net consists of an encoder-decoder segmentation framework (S), reconstruction network (R), and transferblock (T). It takes the advantages of the limited labeled samples. In segmentation framework, we use the residual mechanism to extract high-quality features from the input images and skip operation to improve the processing of the features between the encoder and decoder parts of the network. We present the reconstruction network for reconstructing feature generation in a unsupervised training strategy. The transfer block is used to co-optimization lean the features maps between segmentation framework and reconstruction network. We learn the reconstruction features with that of segmentation network, we embed them into the segmentation network to optimize the model with the mix loss function. COL-Net is trained under the guidance of the mix loss function. As shown in Table 3, the reconstruction network demonstrates its efficacy for improving the segmentation results in the limited labeled stroke medical images. Our study proves useful in mitigating the shortage of labeled samples of penumbra targets, with two dozens of labeled samples proving sufficient to auxiliary train a highly efficient segmentation model.

### 5.2. Loss Functions

Loss function is used to optimize the model by estimating the inconsistency between the predicted value and the real value of the model. There have two sub-loss functions in COL-Net (*loss*_*r*_ and *loss*_*s*_). The *loss*_*r*_ is used to optimize the reconstruction network, which is based on cross-entropy loss (Equation 1). The *loss*_*s*_ is used to optimize the segmentation network, which is based on DC loss (Equation 2). In order to optimize the COL-Net model as a whole, we combine these two loss functions and propose the mix loss function (*loss*_*mix*_) Equation 3. We use λ to weigh the weight of the two-loss functions. We use 5 experiments to get the optimal λ value by setting λ to 0.0, 0.2, 0.4, 0.6, 0.8, and 1.0, respectively. These experiments use the same training and verification samples, which use 80% labeled samples in the training process. The initialization parameters of the model are in section 4.1.

**Figure 5:**
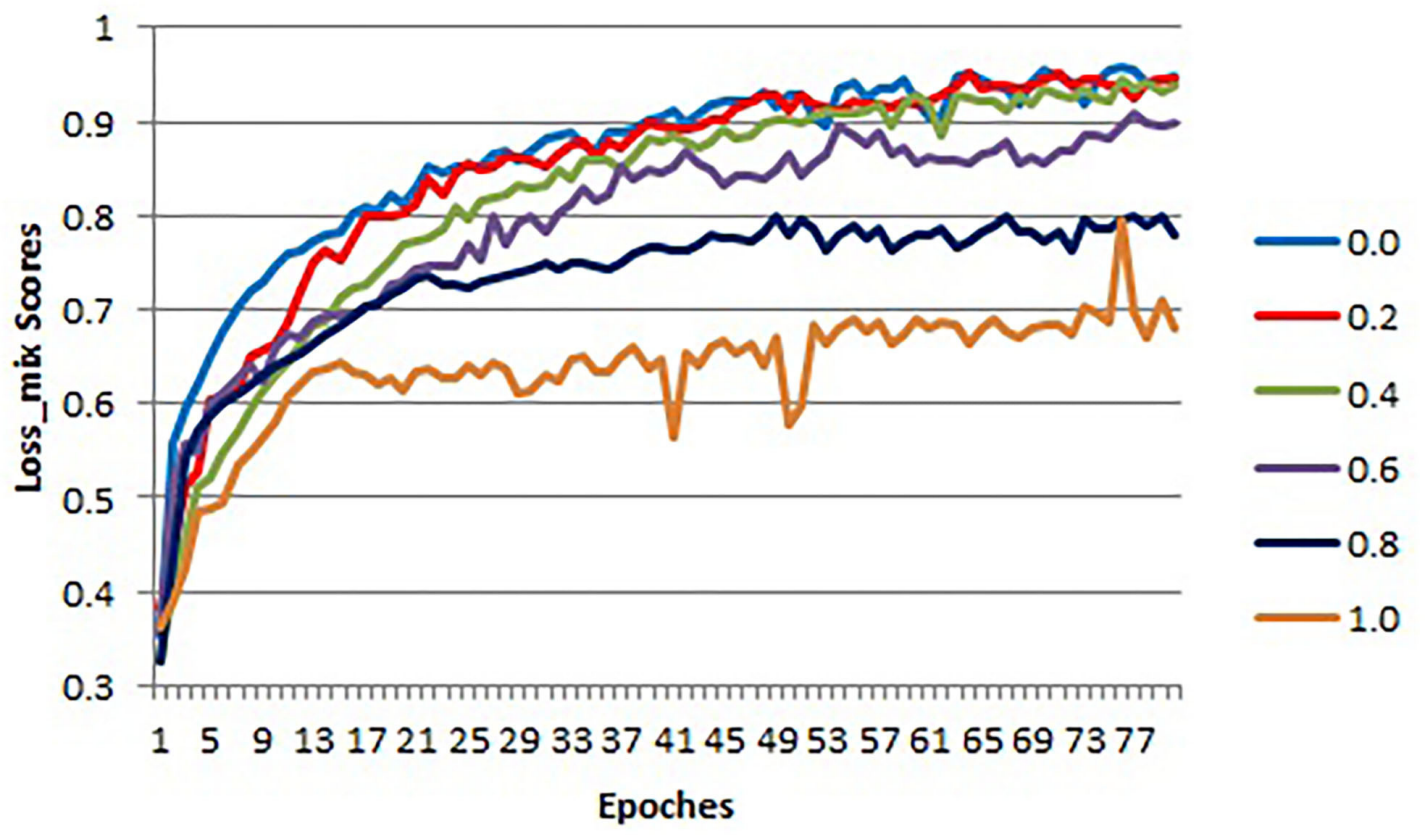
The *loss*_*mix*_ curves of training samples for different λ values.

**Figure 6:**
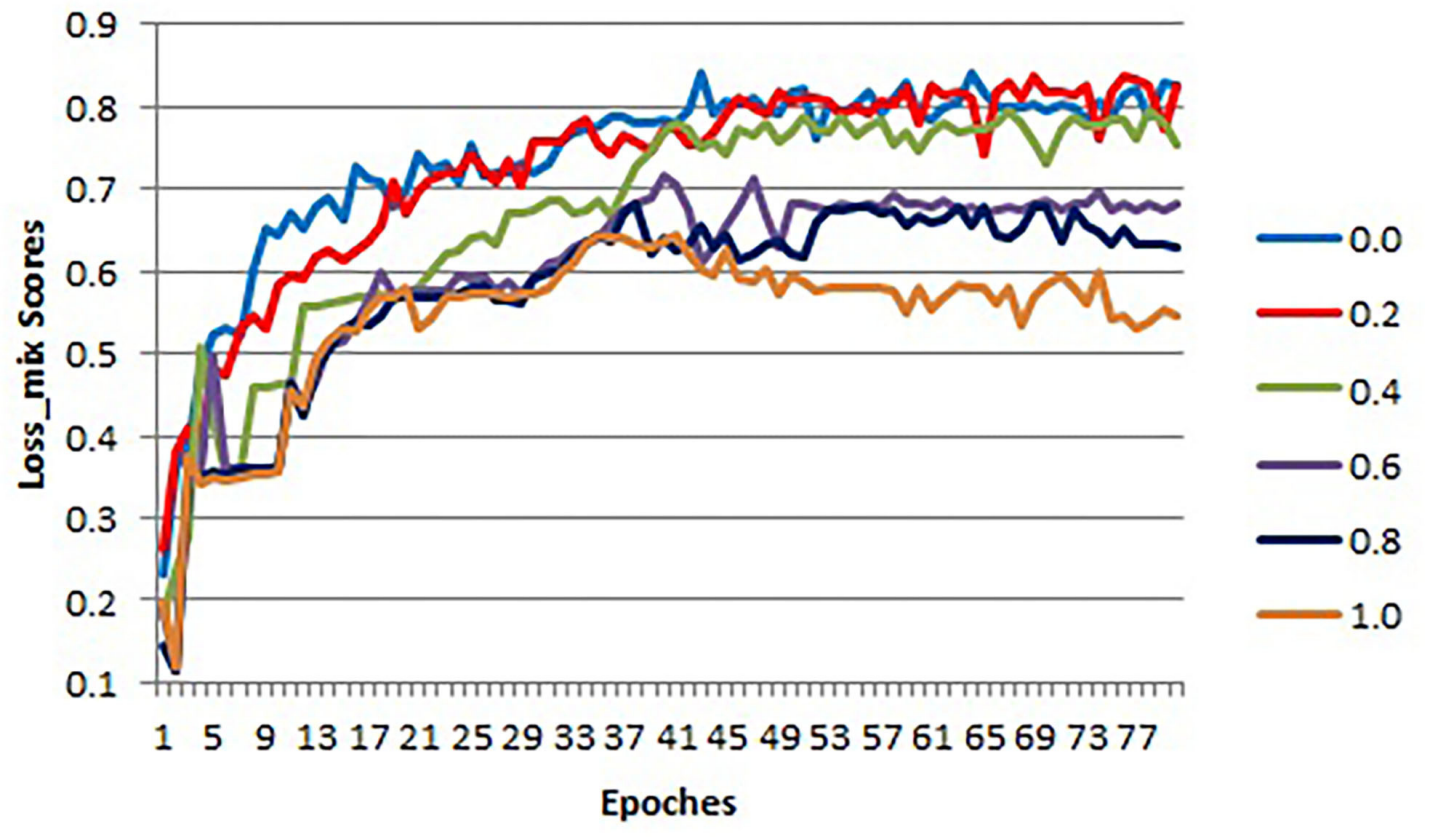
The *loss*_*mix*_ curves of verification samples for different λ values.

In the training and verification processes, we use the *loss*_*mix*_ curves corresponding to *loss*_*mix*_ to measure the quality of λ values. The *loss*_*mix*_ curves of training and verification samples are shown in Figures 5, 6, respectively. In these two coordinates, the horizontal axis corresponds to each epoch value in the training and verification processes respectively, the vertical axis represents the *loss*_*mix*_ score of each epoch. As shown in Figure 5, when λ = 0.0 denotes the reconstruction network (*loss*_*r*_) does not work, we only use the supervised segmentation network (*loss*_*s*_) to training COL-Net. When λ = 1.0 means that the reconstruction network (*loss*_*r*_) and the segmentation network (*loss*_*s*_) have the same weight and influence on COL-Net (*loss*_*mix*_). In general, with the increase of epoch value in the training process, the *loss*_*mix*_ scores of all λ are gradually increasing. In the verification process, with the increase of epoch value, the *loss*_*mix*_ scores of all λ are gradually increasing, except when λ = 1.0. The value of λ is opposite to that of *loss*_*mix*_. When the value of λ increases from 0 to 1.0, the negative effect on *loss*_*mix*_ increases gradually. In the training and verification process, the *loss*_*mix*_ curves corresponding to λ = 0.0 and λ = 0.2 get the best *loss*_*mix*_ score, the coincidence degree is very high, which indicates that the COL-Net method can also achieve the supervised segmentation effect where λ = 0.2. It demonstrates that our proposed method could effective work on the limited labeled samples medical image segmentation task.

### 5.3. Ablation Experiments

To investigate the contributions of the reconstruction network in our proposed method, we conduct several ablation experiments based on COL-Net. In all ablation experiments, we use the training and testing samples as designed in section 4.1. The results of ablation experiments are shown in Table 4. We use the representative U-Net as the baseline. We compare the improved Res-UNet based on U-Net. Res-UNet integrates the residual mechanism into the U-Net network. Consequently, the mean DC, ASSD, and HD values are improved by 1.00%, 1.24 *mm*, and 21.57 *mm* for penumbra tissues segmentation. Compared with the baseline U-Net, the Res-UNet increases the depth of the network and uses a residual mechanism to alleviates the vanishing gradient problem of the deep network. Furthermore, we compare the improved RA-UNet based on the attention mechanism. RA-UNet integrates attention mechanism into skip connection paths of Res-UNet. The main measure means the DC value of RA-UNet is improved by 4.00% than that of the Res-UNet. Compared with the Res-UNet, the attention mechanism in RA-UNet extract higher quality features from the input images, which is more conducive to improve the model's performance. Compared with other supervised models, our proposed COL-Net obtains the top-level DC and ASSD values for penumbra segmentation. The mean DC value of the COL-Net is only 1.0% lower (worse) than that of RA-UNet, the mean ASSD value of the COL-Net is only 0.38 *mm* higher (worse) than that of RA-UNet, the HD value of the COL-Net is 0.74 *mm* lower (better) than that of RA-UNet. Other supervised models use all labeled training samples to fine-tune the models, while our COL-Net model only uses 24 labeled training samples. Although the segmentation performance of the COL-Net model is not the best, it has great advantages to solve the segmentation task with the limited labeled samples.

**Table 4:** Ablation study about performance of 4 networks with DC, ASSD and HD metrics.

**Methods**	**DC**	**ASSD**	**HD**
U-net (Ronneberger et al., 2015)	75.00 (±12.00)	3.34 (±2.18)	64.11 (±29.14)
Res-UNet (He et al., 2016)	76.00 (±9.00)	2.10 (±0.57)	42.54 (±21.14)
COL-Net	79.00 (±8.00)	2.41 (±2.19)	34.20 (±24.05)
RA-UNet (Jin et al., 2018)	80.00 (±3.00)	2.03 (±0.86)	39.43 (±27.07)

*DC:%, ASSD:mm, HD:mm*.

### 5.4. Visualization of the Prediction Results

**Figure 7:**
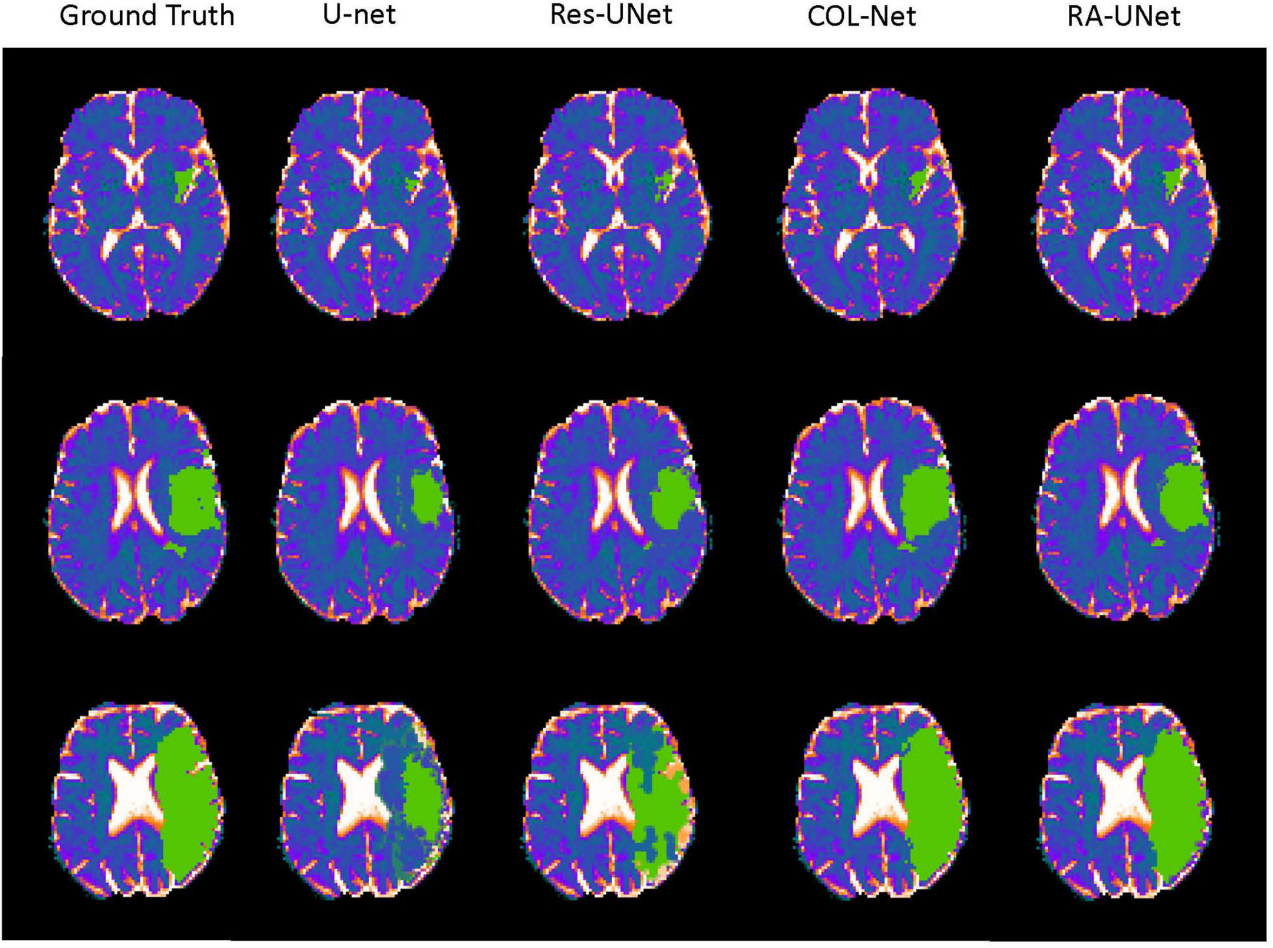
The visualization of the prediction resluts (green regions are the labeled lesions and predicted lesions, respectively).

In order to further analyze the performance of the COL-Net, we conduct several extended experiments to visualize the predicted result of a training sample in SPES. There are 30 training samples in the SPES data set, we use 29 samples to train and adjust the models (U-net, Res-UNet, RA-UNet, and COL-Net), the last one is used as a test sample (The 1 − *th* sample). U-net, Res-UNet and RA-UNet are supervised segmentation methods, COL-Net is a semi-supervised segmentation method. We use the optimal weight of COL-Net trained by 80% labeled samples to test the sample. Figure 7 shows the ground truth against the predicted results produced by these 4 models. There are 3 scenarios in Figure 7. The first row in Figure 7 is an example that has small and trivial size penumbra tissues. The second row in Figure 7 is an example that has medium size penumbra tissues. The third row in Figure 7 is an example that has big size penumbra tissues. In the first row, the ground truth image shows the minuteness and triviality of the annotation label. The supervised U-net and Res-UNet methods are not sensitive to the small lesions, which lead to the fact that the predicted size of the segmentation lesion are much smaller than the ground truth. The prediction result of supervised RA-UNet is the closest to the ground truth. Our semi-supervised COL-Net method is not as good as RA-UNet in predicting small lesions. This is mainly because the attention mechanism in RA-UNet can capture more sensitive feature information under the guidance of a supervised training strategy. In the second and third rows, the green lesion regions are bigger than those in the first row. The prediction results of U-net and Res-UNet methods are still significantly smaller than that in the ground truth. The prediction results of RA-UNet and COL-Net are the closest to ground truth. This shows that the proposed COL-Net model is comparable to the advanced supervised deep learning model in capturing the features of relatively large lesions.

### 5.5. Robustness on SISS Challenge Dataset

In order to further verify the performance of COL-Net, we verify it on SISS challenge. We retain the parameters trained on the SPES dataset, and use the reshape method to scale the size of the SISS dataset image to be consistent with the size of the SPES dataset image. The SISS challenge is a subtask of the ISLES 2015 challenge, which contains 64 samples, including 28 labeled training samples and 36 unlabeled testing samples. We perform the experiment on this data set: we use the pseudo unlabeled samples to train reconstruction network. We use the 80% labeled samples to train segmentation network and all test unlabeled samples on the fine-trained segmentation network. Finally, we upload the prediction results of the testing samples to the challenge web page. The obtained DC, ASSD, and HD are given in Table 5. It shows that, in all methods, COL-Net adopts a semi-supervised training strategy and achieves the average DC, ASSD, and HD to 56.00%, 9.432 *mm*, and 44.02 *mm*. The performance of the proposed COL-Net is second only to the best two supervised segmentation methods (UK-Imp2 and Lianl1), ranking third. This not only shows that COL-Net can approach the performance of the supervised method in the case of a limited labeled samples but also proves that our model has strong generalization ability.

**Table 5:** The results of the participants of the SISS dataset.

**Methods**	**DC**	**ASSD**	**HD**	**Manner**
UK-Imp2	**59.00** (±31.00)	**5.96** (±9.38)	37.88 (±30.06)	Supe
Lianl1	57.00 (±29.00)	8.22 (±16.25)	43.02 (±30.48)	Supe
COL-Net	56.00 (±28.00)	9.432 (±17.05)	44.02 (±45.10)	Semi
Fengc1	55.00 (±30.00)	8.13 (±15.15)	**25.02** (±22.02)	Supe
Martc2	50.00 (±32.00)	14.69 (±17.82)	80.06 (±22.00)	Supe
Abdua1	43.00 (±31.00)	16.85 (±15.71)	74.66 (±25.10)	Supe

*DC:%, ASSD:mm, HD:mm. Semi, Semi-supervised training strategy; Supe, Supervised training strategy*.

## 6. Conclusions

We propose a novel co-optimization learning network (COL-Net). The COL-Net method tries to use a co-optimization learning strategy to train the segmentation model on the basis of the limited labeled samples and the unlabeled samples. It consists of 3 parts: segmentation network (S), reconstruction network (R), and transfer block (T). The mix loss function is proposed to co-optimization COL-Net. It is verified to segment penumbra tissues from brain MRIs on the SPES challenge and an extended SISS challenge. The results demonstrate that COL-Net achieves the state-of-the-art segmentation performance. Extensive experiments show that COL-Net outperforms most supervised segmentation methods and reaches the advanced level on the limited labeled samples. However, the segmentation accuracy of this method on small lesions needs to be improved. In the face of a limited labeled samples, it is a meaningful attempt to use a co-optimization learning semi-supervised method to make segmentation and prediction. In future work, we will explore semi-supervised segmentation methods with better generalization performance.

## Data Availability Statement

The original contributions presented in the study are included in the article/supplementary material, further inquiries can be directed to the corresponding author.

## Ethics Statement

Ethical review and approval was not required for the study on human participants in accordance with the local legislation and institutional requirements. Written informed consent for participation was not required for this study in accordance with the national legislation and the institutional requirements. Ethical review and approval was not required for the animal study because this is a public evaluation benchmark for ischemic stroke lesion segmentation from multispectral MRI (Everyone can obtain the data from http://www.isles-challenge.org/).

## Author Contributions

LL and HZ contributed to conception and design of the study. JZ and J-xW organized the database. LL performed the statistical analysis and wrote the first draft of the manuscript. SX wrote sections of the manuscript. All authors contributed to manuscript revision, read, and approved the submitted version.

## Funding

This work described in this paper was supported by the National Natural Science Foundation of China under grant nos. 61802442, 61772552, 61622213, and 61728211; the 111 project (no.B18059).

## Conflict of Interest

The authors declare that the research was conducted in the absence of any commercial or financial relationships that could be construed as a potential conflict of interest.

## Publisher's Note

All claims expressed in this article are solely those of the authors and do not necessarily represent those of their affiliated organizations, or those of the publisher, the editors and the reviewers. Any product that may be evaluated in this article, or claim that may be made by its manufacturer, is not guaranteed or endorsed by the publisher.

## Footnotes

^1^ www.isles-challenge.org

^2^ www.isles-challenge.org
